# A Study on Measures to Preserve Chlorine and Ammonia Oxygen Removal

**DOI:** 10.3390/ma18061347

**Published:** 2025-03-18

**Authors:** Kecheng Shang, Zhonglin Li, Weiguang Zhang, Yibing Li

**Affiliations:** 1 Department of Materials Science and Engineering, Guilin University of Technology, Guilin 541004, China; shangkecheng0506@163.com (K.S.); zhangwg@glut.edu.cn (W.Z.); 2 Collaborative Innovation Center for Exploration of Nonferrous Metal Deposits and Efficient Utilization of Resources, Guilin University of Technology, Guilin 541004, China; 3 Key Laboratory of New Processing Technology for Nonferrous Metals and Materials, Ministry of Education, Guilin University of Technology, Guilin 541004, China

**Keywords:** graphite anode, manganese anode, zinc sulphate, gas chromatography

## Abstract

Ammonia zinc refining has the benefits of low energy consumption, high zinc recovery, and good environmental protection compared with traditional acid and alkaline zinc refining. However, in the production process of refining zinc with ammonia, the anode undergoes chlorine precipitation, and then the oxidation of the ammonia precipitation of some nitrogen occurs. Ammonia replenishment is a cumbersome process that results in large amounts of ammonia volatilization and environmental pollution. In ammonia zinc refining, it is important to ensure the concentration of ammonia and chlorine, as the graphite anodes used in conventional ammonia zinc refining do not retain chlorine and ammonia and dissolve slowly due to oxidation. Therefore, this paper proposes a new measure to conserve chlorine and ammonia to reduce anode chlorine generation by adding an anionic barrier layer and selecting manganese anode materials with selective oxygen precipitation. Under the conditions of 50 × 100 mm sized electrodes, a current density of 350 A/m^2^, and a temperature of 60 °C, a graphite anode and manganese anode were used for electrowinning and for the collection of anode gas under different additive conditions. For the first time, we present a comparative analysis of gas composition, using gas chromatography to demonstrate the feasibility of the different measures used to preserve chlorine, ammonia, and oxygen for industrial applications, as well as the advantages of using these methods in reducing costs. And the experiments show that, by adding the anionic barrier layer, adding urea, and using manganese anode materials with selective oxygen precipitation, the nitrogen precipitation in the anode gas can be reduced to 40–50%, and oxygen precipitation reaches 48.76%.

## 1. Introduction

As an environmentally friendly metallurgical process, electrolytic, ammonia-leaching, zinc-refining technology has attracted much attention in the field of zinc smelting in recent years due to its advantages, such as the efficient utilisation of low-grade zinc oxide ore and the avoidance of hydrogen sulphide emissions. Its advantages over the traditional sulfuric acid system include the fact that the electrodeposition voltage can be reduced to 2.5–2.8 V (saving energy by 18–26% compared to the sulfuric acid method) and the fact that the electrolyte can be recycled, which reduces the discharge of acidic wastewater [1,2,3]. However, the irreversible depletion of NH_3_ in the anodic zone and the continuous precipitation of Cl_2_ during the electrolysis process leads to difficulty in maintaining the ammonia equilibrium, and every 1 ton of zinc produced needs to be supplemented with 0.5–0.8 tons of ammonia. The resulting loss of ammonia volatilization not only increases raw material costs but also poses the risk of fugitive gases and the eutrophication of water bodies. And the root of the problem is the existence of mutual competition between the OER [4] and CER [5] at high Cl^−^ concentrations (5–7 mol/L). Although the standard electrode potential for the oxygen extraction reaction (OER) (1.23 V) is lower than that for the chlorine extraction reaction (CER, 1.36 V) [6,7], the four-electron transfer process involved in the OER has a higher activation energy barrier. The high-Cl^−^-concentration environment further exacerbates the kinetic dominance of the CER, resulting in the depletion of NH_3_ due to the dominance of Cl_2_ in the anodic product and the initiation of the following side reaction: 4NH_3_ + 3Cl_2_ ⟶ N_2_ + 2NH_4_Cl + 2HCl.

Existing anode material systems generally face a performance–cost–life trade-off dilemma in high-Cl^−^ ammonia media [8,9]. Although graphite anodes have cost advantages, their loose, porous structure makes it difficult to block Cl^−^ penetration. Irreversible corrosion of C + 2Cl_2_ ⟶ CCl_4_ occurs under Cl_2_ oxidation, leading to electrode chalking and electrolyte carbon contamination. A titanium-based IrO_2_ anode can reduce OER overpotential by noble metal catalysis. However, a reversible Cl^−^ adsorption layer is formed on the IrO_2_ surface at high Cl^−^ concentrations, which triggers the preferential precipitation of Cl_2_. The high cost of Ir restricts industrial applications. Although lead-based anodes have better corrosion resistance, the PbO_2_ passivation layer generated on its surface increases the OER’s potential, while lead ion leaching causes the cathode zinc purity to decrease. Defects in these materials can cause the cost of ammonia replenishment to comprise a disproportionately high percentage of the production costs, and they are susceptible to the risk of heavy metal contamination.

In recent years, studies targeting anode-selective modulation have been conducted in low-Cl^−^ systems (e.g., seawater electrolysis, Cl^−^ ≈ 0.5 mol/L). For example, Ling et al. [10] promoted OER activity by optimising the electronic structure of the OER-active site through transition metal doping (Fe, P-Ni Se_2_). Guo et al. [11] inhibited the CER by constructing an alkaline microenvironment on the surface of Cr_2_O_3_-Cox. However, when the Cl^−^ concentration reaches 5–7 mol/L in an ammonia-refined zinc system, conventional physical barriers (e.g., MnO_x_ coatings) undergo lattice distortions due to high osmotic pressures, resulting in a sudden drop in Cl^−^ shielding efficiency. Therefore, it is difficult to utilize the catalytic materials in the low-Cl^−^ system in the ammonia system for zinc refining, and there is an urgent need to develop anode materials that can be adapted to the high-Cl^−^ environment.

Based on this background, this paper proposes a new anode material, MnO_x_-IrO_2_/Ti, and additives for chlorine inhibition. This new anode material is able to synergistically inhibit the precipitation of chlorine gas by the chemisorption of Cl^−^ through the MnO_x_ layer and by a small amount of doped IrO_2_, as well as the effects of chlorine preservation, ammonia preservation [12,13,14,15], and oxygen precipitation in ammonia zinc refining through the addition of an anion and an anionic barrier layer.

## 2. Materials and Methods

### 2.1. Preparation of Experimental Materials and Calculations

All chemical reagents were of analytical grade and procured from Sichuan Xilong Scientific Co., Ltd. (Chengdu, China). The zinc–ammonia–chloride complex electrolyte was synthesized by dissolving analytical-grade zinc oxide (99% purity) in 4 mol/L ammonium chloride solution (99.5% purity), yielding a final Zn(NH_3_)_2_Cl_2_ system with a zinc ion concentration of 50 g/L Zn^2+^. The introduction of polyethylene glycol 8000 (PEG8000) at 600 mg L^−1^ significantly enhances the performance of zinc electrowinning through three synergistic mechanisms: (1) facilitating regular zinc deposition at the cathode by modulating interfacial charge transfer kinetics; (2) maintaining electrolyte uniformity through steric stabilization effects; (3) suppressing particle agglomeration via surface adsorption, ultimately achieving a 12.7% improvement in current efficiency compared to additive-free systems. The electrowinning process was conducted at an industry-standard current density of 350 A/m^2^. Current efficiency (CE) and energy consumption (EC) were determined through continuous potential monitoring between electrodes combined with chronometric quantification of electrolysis duration, as described in Equations (1) and (2) [16,17]:

(1) $$\mathrm{CE}=\frac{\mathrm{nFm}}{3600\mathrm{ItM}}\times 100\%$$

(2) $$\mathrm{EC}=\frac{\mathrm{ITV}}{m}$$

where n denotes the electron transfer number, F = 96,485 C/mol represents the Faraday constant, I (A) is the applied current, t (h) indicates the duration of electrolysis, m (g) corresponds to the actual cathodic zinc deposition mass, M = 65.38 g/mol stands for zinc’s molar mass, and V (V) refers to the average cell voltage across the electrodes.

**Figure 1:**
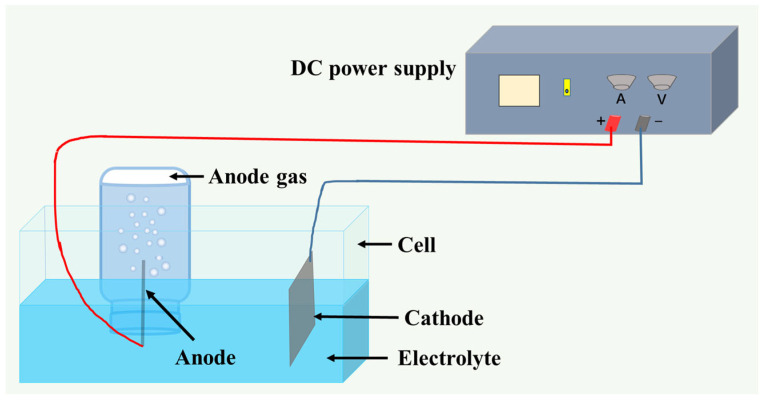
Schematic diagram of the experimental anode gas collection device.

Electrodeposition was conducted in a thermostatically controlled water bath using graphite and MnO_x_-IrO_2_/Ti anodes. The process employed 50 × 100 mm electrodes under standardized conditions: 60 °C operating temperature, 350 A/m^2^ current density, 35 mm interelectrode spacing, and 10 h duration. Triplicate experiments were performed for each additive concentration level, with anodic gases quantitatively collected via the water displacement method. Data were statistically averaged, and the experimental configuration is detailed in Figure 1.

The MnO_x_-IrO_2_/Ti composite anodes were fabricated using 0.5 mm thick titanium sheets (99.9% purity) as substrates. Pretreatment involved sequential surface modifications: (1) ultrasonic degreasing in 5 mol/L NaOH to remove organic contaminants; (2) chemical etching in 1 mol/L oxalic acid at 100 °C for 30 min; (3) plasma-enhanced chemical vapor deposition (PECVD) of a 3 nm Pt interlayer. The active coating was synthesized by dip-coating with a precursor solution containing 50 wt% Mn(NO_3_)_2_, 1 wt% TiO_2_, 2 wt% Na_2_SiO_3_, and 0.02 wt% IrO_2_, followed by multiple coating in a muffle furnace at a decomposition temperature of 200 ℃ and, finally, annealing at 500 ℃.

### 2.2. Experimental Equipment and Material Characterization

Anode gas composition was quantified using a TRACE 1300 gas chromatograph (Thermo Scientific, Karlsruhe, Germany) with manual peak integration. Phase analysis and crystallographic parameters were determined via X’Pert PRO X-ray diffraction (Panalytical, Almelo, The Netherlands). Material surface morphology was characterized by S-4800 field-emission scanning electron microscopy (Hitachi, Tokyo, Japan), while elemental composition was evaluated using an INCA IE 350 energy-dispersive X-ray spectroscopy system (Oxford Instruments, Oxford, UK).

## 3. Results and Discussion

### 3.1. Effect of Graphite Anode and Addition of Sulphate Anion Barrier on Electrolysis

**Figure 2:**
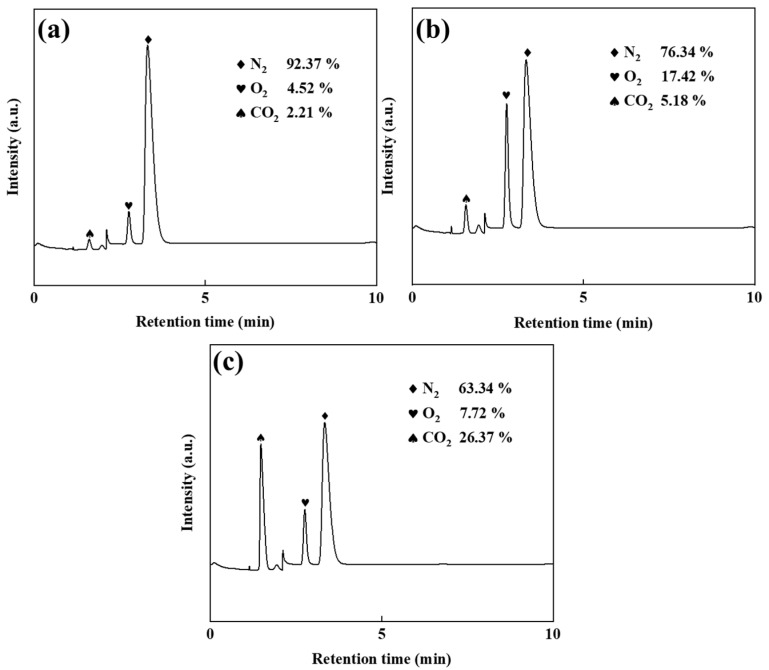
Gas chromatogram diagrams: ZnSO_4_ free (**a**), 10% ZnSO_4_ (**b**), and 20% ZnSO_4_ (**c**).

The electrolyte maintained a total Zn^2+^ concentration of 50 g/L, with Zn(NH_3_)_2_Cl_2_ solutions containing 45 g/L and 40 g/L Zn^2+^. ZnSO_4_ additions of 5 and 10 g/L (corresponding to 10% and 20% of the total zinc concentration, respectively) were applied. Electrolysis experiments employed graphite anodes to collect anode gases, and gas chromatography analysis (Figure 2) revealed the influence of ZnSO_4_ supplementation on gas composition.

Figure 2a reveals, through gas chromatographic analysis, that the anode gas in ZnSO_4_-free electrolysis primarily consists of N_2_ (92.37%), with minimal O_2_ content (4.52%). This composition demonstrates a two-stage anodic mechanism: initial Cl^−^ oxidation through electrochemical discharge, followed by redox interactions with ammonia species (NH_3_/NH_4_^+^). The suppressed oxygen evolution further indicates the limited participation of hydroxide ions (OH^−^) or water molecules in the oxidation processes. As shown in Figure 2b, the addition of ZnSO_4_ significantly reduced the anode gas nitrogen content (76.34%) compared to no addition of ZnSO_4_. The reduction in Cl_2_ emissions further confirms that ZnSO_4_ inhibits the chlorine oxidation at the anode, thus limiting the chlorine evolution and subsequent ammonia displacement reaction. Concurrently, the O_2_ content rose to 17.42%, demonstrating that suppression of Cl^−^ oxidation enhanced OH^−^/H_2_O discharge at the anode, thereby elevating gas-phase oxygen generation. Comparative analysis of Figure 2a,b reveals a CO_2_ content increase from 2.21% to 5.18%, with graphite serving as the exclusive carbon source in the system. This confirms the enhancement of anodic graphite oxidation under ZnSO_4_ addition, evidenced by the concomitant rise in gas-phase CO_2_ generation and accelerated carbon corrosion kinetics. As shown in Figure 2c, the anodic N_2_ content decreased further to 63.34% upon introducing 20% ZnSO_4_, demonstrating suppressed Cl^−^ oxidation at the anode. Concurrently, the O_2_ level declined to 7.72%, suggesting sulphate-enhanced carbon oxidation in graphite [18,19,20], which elevated CO_2_ generation while reducing residual N_2_ proportion.

**Figure 3:**
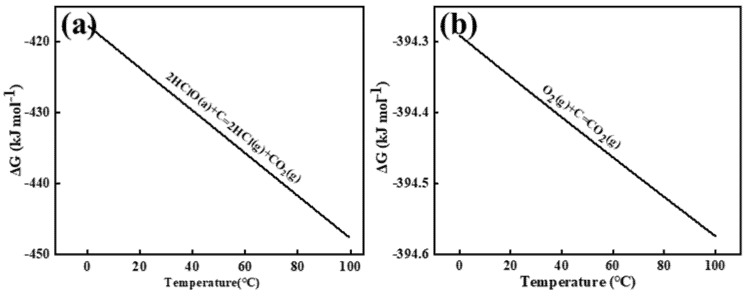
The Gibbs free energy of HClO (**a**) and O_2_ (**b**) reacts with C in an aqueous solution. a and g in the diagram represent the states of the reactants, with a—representing the ionic form and g—representing the gaseous form.

The Gibbs free energy was calculated using the relationship ΔG = −RT ln K_c_, where R represents the molar gas constant (8.314 J·mol^−^¹·K^−^¹), T denotes absolute temperature, and K_c_ is the equilibrium constant. Figure 3 illustrates the Gibbs free energy changes for the electrochemical reactions between chlorine (Cl_2_), oxygen (O_2_), and carbon (C) in aqueous solution, specifically those occurring at the graphite anode interface. As shown in Figure 3a,b, chlorine gas induces gradual oxidation of the graphite anode carbon to CO_2_. In aqueous solution, anodically generated Cl_2_ undergoes rapid hydrolysis near the interface (Cl_2_ + H_2_O ⇌ HClO + Cl^−^ + H^+^ [13]), resulting in hypochlorous acid (HClO) serving as the primary reactive species. HSC 6.0 calculations of Gibbs free energy changes confirm the spontaneity of both reactions. Comparative analysis reveals that oxygen species on the aqueous-phase anode surface exhibit enhanced reactivity, demonstrating faster reaction kinetics with carbon and consequently accelerated graphite consumption.

**Figure 4:**
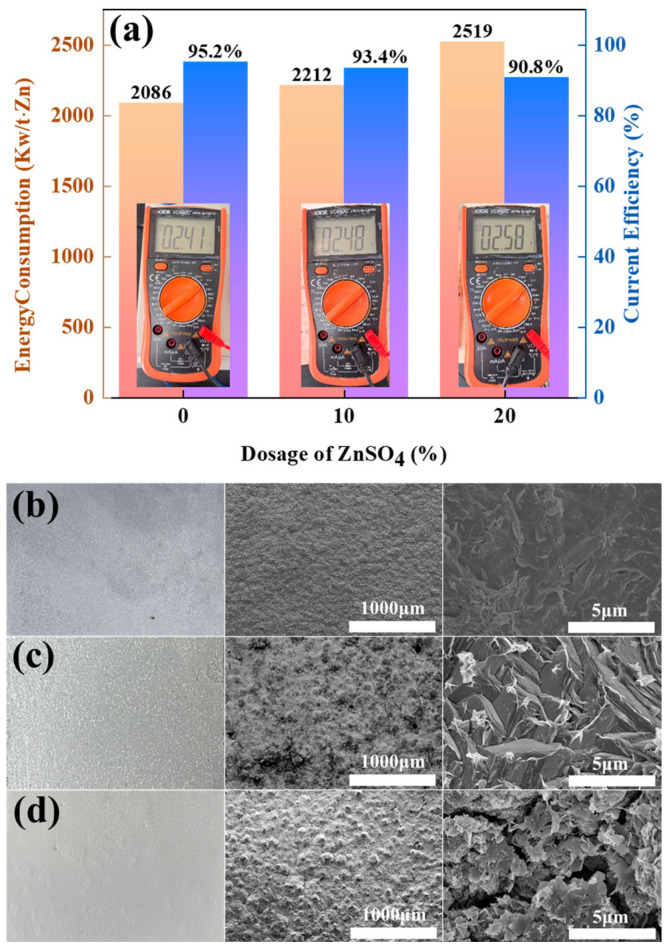
(**a**) Energy consumption and CE of ZnSO_4_-free, 10% ZnSO_4_ addition, and 20% ZnSO_4_ addition conditions; (**b**) macroscopic diagrams of cathode zinc under ZnSO_4_-free, (**c**) 10% ZnSO_4_ addition, and (**d**) 20% ZnSO_4_ addition conditions.

To evaluate the impact of ZnSO_4_ addition on zinc electrowinning performance in the Zn-NH_3_-Cl system, current efficiency (CE) and DC energy consumption were systematically analysed under identical operational parameters. Figure 4 quantitatively demonstrates how ZnSO_4_ supplementation modulates three key metrics: cathodic zinc deposition dynamics, DC electrolysis efficiency, and specific energy expenditure.

Figure 4a shows that the current efficiency (CE) of the amino-hybrid acid system modified with ZnSO_4_ remains above 90%. Notably, the baseline system without ZnSO_4_ achieved higher current efficiency (95.2%) and lower energy consumption (2086 kW h/t Zn). However, the introduction of 20% ZnSO_4_ significantly increased the energy consumption to between 2212 and 2519 kW h/t Zn, which was attributed to the sulphate-induced voltage increase during electrolysis. This arises from sulphate ions (SO_4_^2−^) exhibiting lower ionic mobility than chloride ions (Cl^−^), consequently elevating solution resistance. Figure 4b–d reveal that zinc cathodes deposited under all three conditions exhibit macroscopically smooth and dense morphologies. Notably, ZnSO_4_-supplemented systems yield off-white zinc flakes, distinctively lighter than the greyish-cyan deposits formed without ZnSO_4_. Electron microscopy further demonstrates that the ZnSO_4_-free zinc deposits maintain a fully dense microstructure devoid of detectable porosity. At 10% ZnSO_4_ supplementation, zinc deposits adopt a microstructure of tightly packed cubic blocks with observable intergranular micropores. Elevating ZnSO_4_ concentration to 20% fundamentally alters crystallization pathways, resulting in loosely stacked nanoflake architectures with extensive cleavage planes.

Therefore, industrial-grade graphite can be used as a functional anode. Gas composition analysis confirmed that the addition of ZnSO_4_ can effectively inhibit the evolution of Cl_2_, but excessive addition (≥20%) leads to a decrease in current efficiency along with a significant increase in energy consumption. In addition, the accelerated degradation of graphite anodes under sulphate-enhanced conditions was experimentally verified.

### 3.2. Manganese Anodes and Their Effect on Electrolysis

#### 3.2.1. Manganese Anode Characterization

**Figure 5:**
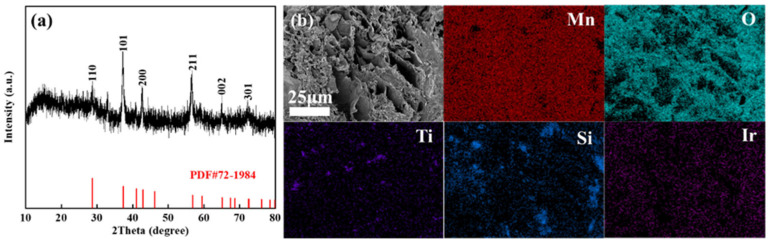
XRD pattern (**a**), SEM (**b**), and corresponding element distribution of the prepared manganese anode.

The XRD pattern of the MnO_x_ anode (Figure 5a) demonstrates low crystallinity, with thermally decomposed manganese oxides showing faint diffraction peaks. Alignment with the β-MnO_2_ reference (PDF#72-1984) confirms weak signals only at the (110), (101), (200), and (211) crystallographic planes. A prominent broad diffraction peak centred at 2θ ≈ 20° further indicates the coexistence of amorphous phases, suggesting that the synthesized MnO_x_ primarily consists of low-crystallinity manganese oxides with significant amorphous content. SEM characterization reveals the MnO_x_ anode exhibits a lamellar porous architecture decorated with polydisperse particulate features. This lamellar porosity yields an expanded surface area configuration, beneficial for boosting the electrode’s electrocatalytic activity. EDS mapping of the MnO_x_ anode confirms the coexistence of Mn, O, Ti, Si, and Ir with distinct distribution patterns. Mn, O, and Ir demonstrate homogeneous dispersion, while Ti and Si exhibit localized clustering—a phenomenon attributed to the particulate nature of titanium and silicon additives undergoing agglomeration during synthesis. Surface-enriched Ti facilitates the formation of an oxyphilic oxide layer, thereby improving interfacial reaction kinetics, while the uniformly distributed Ir enhances bulk electrical conductivity and promotes oxygen evolution reaction (OER) activity.

In order to determine the valence distribution of various major elements in the prepared manganese oxide anode catalytic materials, X-ray photoelectron spectroscopy (XPS) was used to detect and analyse the prepared materials.

**Figure 6:**
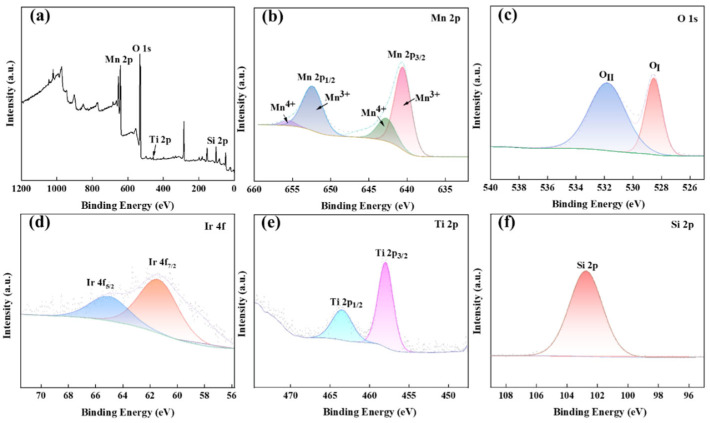
XPS diagram of the prepared MnO_x_ anode: Full spectrum (**a**), Mn 2p (**b**), O 1s (**c**), Ir 4f (**d**), Ti 2p (**e**), Si 2p (**f**).

Figure 6 presents the XPS analysis of the MnO_x_ anode catalytic material. The survey spectrum (Figure 6a) confirms the presence of Mn, O, Ti, Si, and Ir, with elemental valence states determined through binding energy analysis. Distinct peaks are observed at 653.3 eV (Mn 2p), 529.6 eV (O 1s), 459.1 eV (Ti 2p), and 102.92 eV (Si 2p), while the Ir 4f signal is absent due to the low IrO_2_ loading. Figure 6b–f, respectively, display the high-resolution spectra of Mn 2p, O 1s, Ti 2p, Si 2p, and Ir 4f. The Mn 2p spectrum exhibits spin–orbit splitting into 2p3/2 and 2p1/2 components, with the Mn 2p3/2 peaks at 641.05 eV and 642.45 eV corresponding to Mn^3+^ and Mn^4+^ oxidation states, respectively [21,22].

The Mn 2p1/2 core level exhibits spin–orbit split components at 652.85 eV and 656.15 eV, assigned to Mn^3+^ and Mn^4+^, respectively [23], confirming the coexistence of MnO_2_ and Mn_2_O_3_ phases. The Mn^3+^/Mn^4+^ redox pair exhibits strong adsorption capacity for oxygen intermediates, facilitating O-O bond formation and enhancing oxygen evolution reaction (OER) activity. Deconvolution of the O 1s spectrum reveals two dominant peaks at 528.65 eV (Me-O bonds) and 531.8 eV (Si-O bonds) [24]. The 531.8 eV peak’s higher intensity indicates abundant oxygen vacancies in the thermally synthesized MnO_x_ [25,26,27], which promote OER kinetics. Ir 4f analysis shows doublet peaks at 62.55 eV (4f7/2) and 64.8 eV (4f5/2) [28], suggesting IrO_2_ formation. Titanium-supported Ir sites effectively reduce OER overpotential while suppressing chloride ion oxidation. The Si 2p spectrum exhibits a single peak at 102.75 eV, characteristic of Si-O bonding [29]. The Ti 2p spectrum displays characteristic spin–orbit splitting with 2p3/2 and 2p1/2 peaks at 458.1 eV and 463.75 eV, confirming TiO_2_ formation [30].

#### 3.2.2. Manganese Anode Gas Characterization

**Figure 7:**
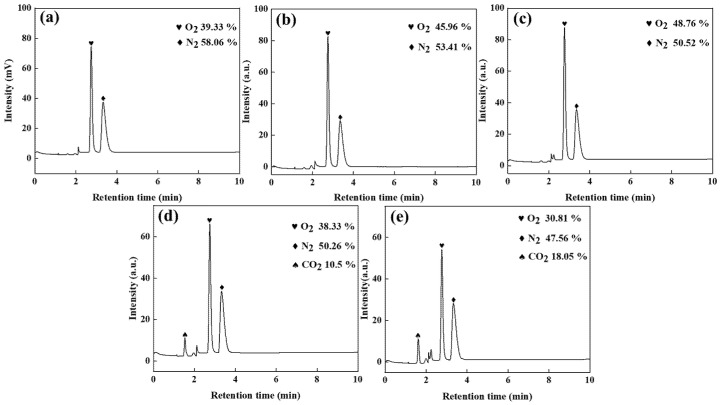
Gas chromatogram of manganese anode gas: (**a**) zinc-sulphate-free, (**b**) 20% zinc sulphate addition, (**c**) 30% zinc sulphate addition, (**d**) 20% zinc sulphate and 5 g/L urea addition, and (**e**) 30% zinc sulphate and 5 g/L urea addition conditions.

Figure 7 displays gas chromatography profiles of anode off-gases generated during electrolysis with the synthesized MnO_x_ anode under varied operational parameters, where gas sampling was systematically conducted via water displacement methodology.

As shown in Figure 7a, the oxygen and nitrogen contents in the anode gas produced by electrolysis without ZnSO_4_ addition are 39.33% and 58.06%, respectively. In contrast, when graphite is used as the anode, the oxygen content drops to 4.52%, with nitrogen rising to 92.37%, demonstrating the manganese anode’s critical role in promoting oxygen-selective evolution in high-chloride solutions. To suppress chlorine evolution and mitigate ammonia oxidation, adding 20% and 30% ZnSO_4_ elevates oxygen content to 45.96% and 48.76%, respectively, while reducing nitrogen to 53.41% and 50.52%. This occurs because sulphate ions accumulate on the anode surface under electric fields, forming a barrier that inhibits chloride ion oxidation—a mechanism consistent with graphite anode observations. Comparing Figure 7a–c reveals that 20% ZnSO_4_ addition increases oxygen content to 45.96%, whereas 30% ZnSO_4_ provides only a marginal 2.8% further increase (45.96% → 48.76%) and reduces nitrogen by merely 2.89% (53.41% → 50.52%). Therefore, 20% ZnSO_4_ is identified as the optimal concentration.

The introduction of N_2_H_4_CO moderately reduces nitrogen concentration while simultaneously decreasing oxygen content. This demonstrates that N_2_H_4_CO preferentially undergoes oxidation over ammonia, accelerates CO_2_ evolution kinetics, and reveals the dual nitrogen origin in anode gas: partial oxidation of N_2_H_4_CO and residual ammonia decomposition. Comparative analysis of Figure 7d,e confirms that optimized N_2_H_4_CO addition achieves minimal anode nitrogen levels at 47.56%.

**Figure 8:**
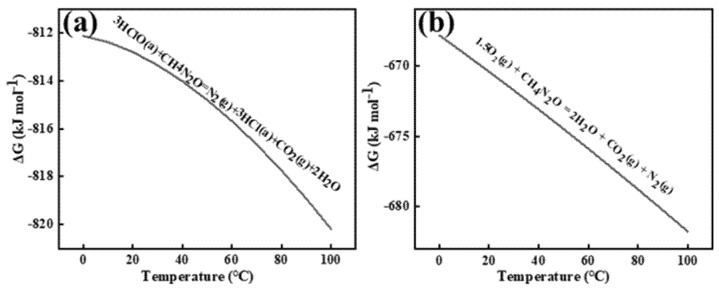
Reaction of urea with hypochlorous acid (**a**) and oxygen (**b**). a and g are used to distinguish between types of states of matter.

Figure 8 shows the Gibbs free energy changes for the two reactions occurring near the anode interface under this condition, as calculated by HSC 6.0.

Prior studies confirm that electrogenerated chlorine undergoes rapid hydrolysis in aqueous media to form hypochlorous acid (HClO), establishing HClO as the primary oxidative agent for urea decomposition. Electrochemical analysis reveals that N_2_H_4_CO competitively reacts with both anode-derived HClO and dissolved oxygen species. The kinetic preference for HClO-mediated oxidation stems from its stronger oxidizing capacity and inherent oxidative instability compared to molecular oxygen.

#### 3.2.3. Effect of Manganese Anodes on Zinc Cathodes

**Figure 9:**
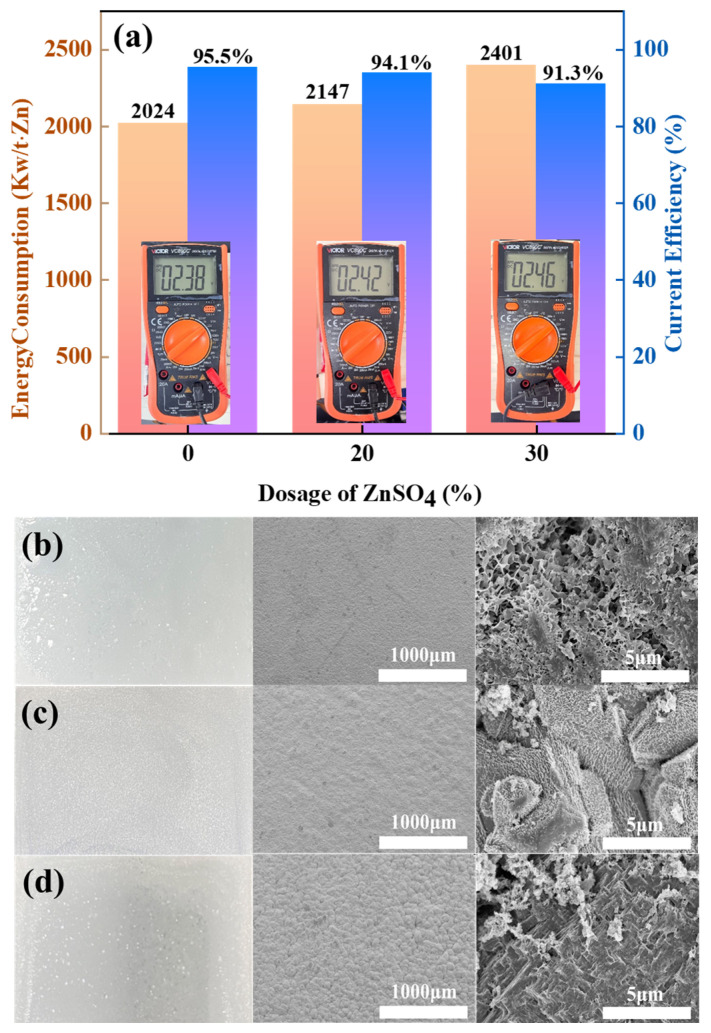
Difference in energy consumption and current efficiency between ZnSO_4_-free and 20% ZnSO_4_ and 30% ZnSO_4_ added conditions (**a**); macro- and micrographs of zinc cathode without ZnSO_4_ added (**b**); macro- and micrographs of zinc cathode with 20% ZnSO_4_ added (**c**); macro- and micrographs of zinc cathode with 30% ZnSO_4_ added (**d**).

Electrolysis using prepared MnO_x_ anode plates was performed to evaluate the manganese anode’s effects on three critical parameters: zinc deposition at the cathode, DC energy consumption, and current efficiency (CE), with corresponding results detailed in Figure 9.

Direct voltage measurements show that the MnO_x_ anode has a slightly lower electrolysis voltage and a slightly higher current efficiency compared to the graphite anode. Following the same trend as the graphite anode, the CE gradually decreases with increasing ZnSO_4_ concentration, along with a corresponding increase in DC energy consumption. Importantly, the excellent oxygen evolution activity of the MnO_x_ anode maintains a CE of 91.3% at 30% ZnSO_4_ load while consuming 4.7% less energy than graphite under the same operating parameters. The zinc cathode yields macroscopically flat and compact metallic deposits. Microscopic analysis reveals that under ZnSO_4_-free conditions, zinc adopts nanosheet morphology akin to graphite anode growth patterns. However, ZnSO_4_ supplementation induces crystalline restructuring, producing densely packed bulk zinc agglomerates. Conventional PbO_2_ anodes in amine-zinc-refining systems have three key limitations: (1) susceptibility to corrosive degradation, which can lead to leaching of lead ions and affect cathode zinc purity; (2) risk of environmental contamination from heavy metal release; and (3) inherently high oxygen evolution overpotential. In contrast, manganese oxide (MnO_x_) anodes have excellent corrosion resistance and do not undergo toxic metal dissolution, thus effectively maintaining the quality of cathode deposits. In addition, manganese oxide has a lower material cost than PbO_2_ and can be strategically doped to achieve tuneable oxygen evolution kinetics—both lowering the overpotential and increasing the electrolytic efficiency. These combined advantages make manganese oxide the technically and economically optimal anode choice for ammonia-based zinc refining.

## 4. Investigation on the Mechanism of Chlorine Inhibition by Graphite and Manganese Anode

**Figure 10:**
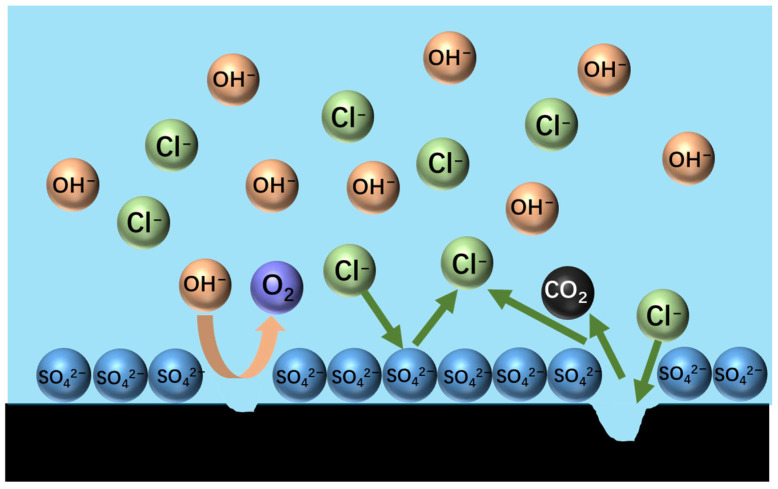
Diagram of sulphate forming a barrier layer on the surface of a graphite anode.

The anode gas consisted mainly of N_2_, which indicated that the graphite anode had minimal selectivity for chlorine evolution during ammonia electrolysis, and the O_2_ content was extremely low. When SO_4_^2−^ was introduced, the O_2_ content rose, suggesting that sulphate-induced competitive adsorption hindered chlorine oxidation. Meanwhile, the increase in CO_2_ content (Figure 10) provides direct evidence of the accelerated oxidative degradation of graphite via the sulphate-mediated corrosion pathway.

The elevated CO_2_ levels primarily result from accelerated carbon oxidation at the anode surface driven by enhanced oxygen evolution. Although chlorine (Cl_2_) generation predominates on graphite anodes, secondary hypochlorous acid (HClO) formation oxidizes residual carbon to CO_2_. Experimental observations revealed sulphate-induced graphite degradation, evidenced by increasing anode material loss as fine particulates. Post-electrolysis analysis showed substantial black graphite powder accumulation at the electrolyzer base, particularly pronounced in 20% ZnSO_4_-supplemented systems.

**Figure 11:**
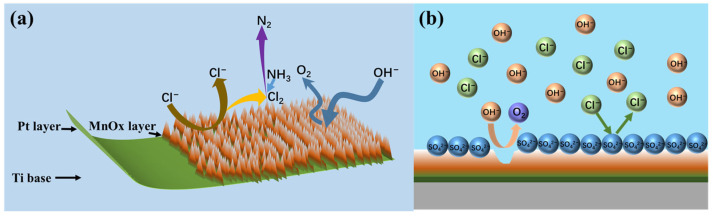
Selective OER by MnO_x_ anode (**a**) and schematic diagram of anion barrier layer (**b**).

**Figure 12:**
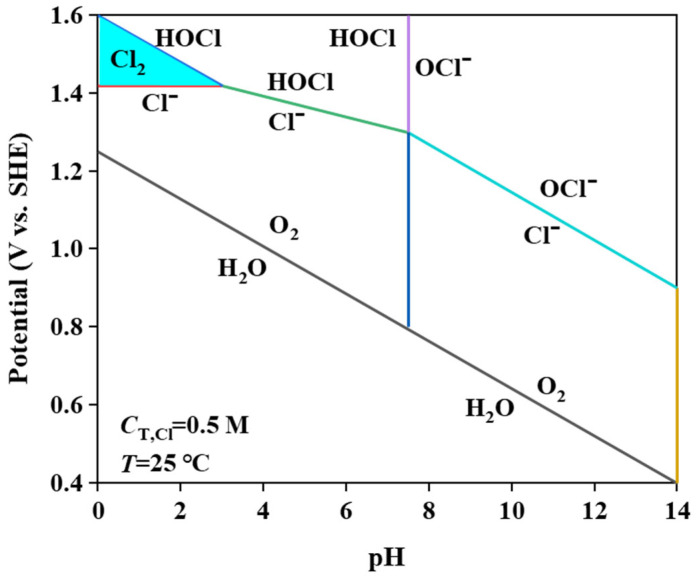
Pourbaix diagram of brine electrolyte.

MnO_2_ and Mn_2_O_3_ constitute the primary catalytic phases in MnO_x_ anode materials. The MnO_6_ octahedral framework serves as the electrochemically active site, where the octahedral coordination geometry dictates both oxygen evolution reaction (OER) activity and structural stability. As evidenced in prior studies [31], the MnO_6_ octahedron undergoes dynamic surface reconstruction by releasing an oxygen atom to form MnO_5_ moieties that bind hydroxyl groups (-OH). Concurrently, the layered architecture of MnO_x_ enables selective ion permeation, exclusively permitting H_2_O and -OH transport through its interlamellar channels. Although IrO_2_ doping effectively reduces OER overpotential, its extreme catalytic activity creates kinetic disparity: IrO_2_-driven OER rates exceed proton diffusion rates by >3 orders of magnitude. This kinetic imbalance induces localized alkaline microenvironment formation at the anode interface, thereby autocatalytically enhancing OER performance through hydroxide concentration effects. Previous studies have shown that the catalytic performance of MnO_x_ can be improved by strategic modifications, including surface defect engineering, metal ion doping, carbon-based hybridization, and Mn^3+^/Mn^4+^ ratio optimization [32,33,34]. In agreement with these reports, our experimental results confirm that the synthesized MnO_x_ anode has a selective oxygen evolution capability when electrolyzed in a highly chlorinated environment. As shown in Figure 11a, some of the chloride ions are discharged at the anode interface, producing Cl_2_, which subsequently reacts with NH_3_ by displacement to produce N_2_ gas. The other part is released as OH^−^ after discharge. Figure 11b shows that the SO_4_^2−^ anion competitively adsorbs on the anode surface, thereby impeding chloride oxidation and further enhancing oxygen precipitation. Notably, Cl_2_ produced by electrolysis is rapidly hydrolysed to form HClO, which dissociates protons (H^+^) that accumulate near the electrode interface. At the same time, the oxidation of hydroxide (OH^−^) produces additional protons that synergistically form an acidic microenvironment in the anode region. Figure 12 [4,11,35] is a Pourbaix plot for chlorides based on previous studies where solution pH, applied potential, and temperature are considered in complex chloride electrooxidation systems. The plot shows that chloride oxidation dominates at pH values below 3.0. The acidified interfacial layer at the anode (pH less than 3.0) will raise the thermodynamic potential of the oxygen evolution reaction (OER), as indicated by the Equation 2Cl^−^ − 2e^−^ = Cl_2_; thus, it thermodynamically facilitates the chloride evolution reaction (CER) through the electrochemical series of substitutions.

(3) $$E_{02}=1.23+\frac{RT}{F}log{\left(a_{H+}\right)}_{anode}+\frac{RT}{4F}logPO_{2}$$

While employing MnO_x_ anode materials for electrolysis, the incomplete formation of MnO_6_ octahedral configurations and Mn^3+^-active sites limits their catalytic selectivity. This structural imperfection permits partial OH^−^ oxidation for oxygen evolution; yet, it cannot fully suppress the competitive chloride oxidation that generates Cl_2_. Consequently, the anode gas composition invariably contains chlorine species alongside oxygen, as evidenced by quantitative gas analysis.

## 5. Conclusions

The amorphous MnO_x_ anode exhibits selective oxygen evolution in a chloride-rich environment, with 48.76% O_2_ content in the anode gas through its active Mn-O coordination site. The application of an anionic barrier reduces the N_2_ content to 47.56%, with the residual N_2_ coming exclusively from the decomposition of NH_3_. This dual strategy—deployment of a manganese-based anode combined with ion migration inhibition—effectively minimizes ammonia/ammonium oxidative substitution, thereby maintaining the initial ammonia concentration during prolonged electrolysis. The MnO_x_-IrO_2_/Ti composite anode developed in this paper has significant potential for industrial applications in high Cl^−^ ammonia electrolytes. Although the initial cost of MnO_x_-IrO_2_/Ti will be about 3–5 times higher (mainly originating from Ir doping), it can achieve long-term cost-saving through the lower CER current efficiency, reduced ammonia replenishment, and longer lifetime. In addition, MnO_x_-IrO_2_/Ti anode avoids the risk of heavy metal contamination of lead-based materials, improves cathode zinc purity, and has significant premium space against the backdrop of tightening environmental regulations. For future research, the main focus should be on the following three aspects: firstly, the development of non-precious metal alternatives to increase OER activity by doping with non-precious metals; secondly, coating durability is optimized and in situ growth and other methods are used to improve the coating bond strength, thus extending the life of the material; finally, monitoring of coating pore changes by means of electrochemical impedance spectroscopy, etc., reduces the chances of sudden failures leading to production stoppages, etc. The above approaches can be used to promote the upgrading of the zinc-refining process in the ammonia system.

## Data Availability

The original contributions presented in the study are included in the article, further inquiries can be directed to the corresponding authors.
